# Recurrent respiratory papillomatosis: role of bevacizumab and HPV vaccination. A literature review with case presentations

**DOI:** 10.2478/raon-2025-0010

**Published:** 2025-02-27

**Authors:** Silvio Sporeni, Francesca Rifaldi, Irene Lanzetta, Ilaria Imarisio, Benedetta Montagna, Francesco Serra, Francesco Agustoni, Paolo Pedrazzoli, Marco Benazzo, Giulia Bertino

**Affiliations:** 1 Department of Internal Medicine and Medical Therapy, University of Pavia, Pavia, Italy; 2 Department of Oncology, Hospital IRCCS Policlinico San Matteo Foundation, Pavia, Italy; 3 Department of Otolaryngology, Hospital IRCCS Policlinico San Matteo Foundation, Pavia, Italy; 4 Department of Clinical, Surgical, Diagnostic and Pediatric Sciences, University of Pavia, Pavia, Italy

**Keywords:** oral cavity papillomatosis, respiratory recurrent papillomatosis, multimodal treatment, systemic therapy, vaccine immunization

## Abstract

**Background:**

Recurrent respiratory papillomatosis (RRP) is a condition caused by human papilloma virus (HPV) infection. Curative treatments aren’t identifiable, and conservative surgery is often the best option to preserve respiratory functions. To date monoclonal antibodies are considered to be a treatment choice with both good efficacy and safety profile.

**Materials and methods:**

A web-based search of MEDLINE/PubMed library from 2000 to 2024 of English-language papers was performed to identify articles by using “respiratory or laryngeal papillomatosis” and “HPV respiratory infection, papillomatosis treatment, papillomatosis vaccine immunization, papillomatosis systemic treatment”. Furthermore, a manual screening of references from original articles was done to identify additional studies. We selected 34 articles.

**Results:**

Since 2009, the systemic administration of Bevacizumab has been used to treat RRP not responding to surgical treatment. The efficacy of an anti-VEGF monoclonal antibody in RRP lesions can be related to their vascular nature. The major concern is the rebound papilloma growth within the cessation of treatment. An interesting solution could be the concomitant use of immunotherapy to both reduce the burden of residual disease and activate the immune system against the HPV-infected cells.

**Conclusions:**

Bevacizumab has a safe profile with a short-term local eradication of HPV. Further prospective research with long-term follow-up is needed to better define its safety and results against the disease recurrence. Considering the role of the anti-HPV vaccine, both, in the prophylaxis of the infection and in the adjuvant setting, the actual data underline the need for evaluation of its therapeutic efficacy for the management of RRP.

## Introduction

Recurrent respiratory papillomatosis (RRP) describes a morbid benign condition caused by the infection of the upper aerodigestive tract operated by human papillomavirus (HPV), a non-encapsulated, double chain icosahedral structured virus composed of 72 capsomeres.

There is currently no curative treatment for RRP. The primary approach consists in surgical excision to debulk the papilloma and ensure an adequate vocal outcome, as much as possible.

### HPV

The term “Papillomatosis” collects a heterogeneous group of non-oncological lesions that affect the mucosal tissue of the oral cavity and upper respiratory tract.

The etiopathogenesis of this morbid condition isn′t totally understood yet, although some potential risk factors were identified: mostly the same that could potentially lead to the develop of Squamocellular Carcinoma (SCC) and can be classified in non-viral and viral risk factors. Excessive smoking, chronic alcoholism, poor oral hygiene, edentations and mucosal trauma inducted by incorrect prosthetic works are fundamental in producing a persistent inflammation and irritation.^1^ Nowadays, especially with the improvement of diagnostic molecular biology techniques, human papillomavirus (HPV) is identified as essential cause of papillomatous lesions.^2^

HPV is a non-enveloped double-stranded circular genome DNA virus classified into Papillomaviridae family, whose 200 different genotypes are known. Up to 90% of the infections are related to genotypes 6 and 11, which are characterized by a weak potential of malignant transformation. The remaining 10% of infections are caused by genotypes 16, 18, 31 and 33, which are associated to a high carcinogenic power and can induce the development of malignancies such as squamous cell carcinoma of the oropharynx, cervix, vagina, uterus, anus and penis.^3,4^ The viral infection is globally widespread, nevertheless with a geographical linked incidence variability. It′s higher in East African countries, due to the underdeveloped economic system and the inefficacy of the medical system in promoting the vaccination program, while the rate in West Asia is the lowest. The transmission can be sexual or non-sexual and, in 1% of general population, HPV 6 and 11 can be “commensal” of the oral cavity, especially in the larynx.^5^ The virus infiltrates the mucosal basal membrane and the profound cell layer, through epithelial discontinuation, where is able to multiply; all the process is mainly driven by genes E6 and E7, key regulators of cell cycle progression.^6^

In order to sustain its proliferation, HPV maintains itself inside the host cell, fusing its genes with the host cell genome: this phenomenon is probably responsible for a persistent viral infection defined as Recurrent Respiratory Papillomatosis (RRP).^7^

### Recurrent Respiratory Papillomatosis (RRP)

Is a rare pathological condition that usually primary affects the upper aerodigestive tract.^8^ Three peaks of onset are recognized at ages 7, 35, and 64.^9^ The juvenile form (JoRRP) is more aggressive, and it′s estimated to affect 4.^3^ per 100000 children, otherwise the adult form (AoRRP) involves 1.8 per 100000.^5,10^ it′s known a geographical as previously said, even if, interestingly, the incidence of RRP is similar in both developed and developing countries.^11^

### Diagnosis

Starting from the larynx, the infection can spread to extra laryngeal structures such as trachea, oropharynx, nasopharynx, nose, oral cavity, and rarely the lung. The involvement of these anatomical structure explains the most frequent symptoms onset: hoarseness, typical of young age, and dysphonia, common in the adults′ forms. Dyspnoea, chronic cough, recurrent respiratory infections, pneumonia, acute respiratory distress, dysphagia are usually result of an upper airway involvement.^12^

Clinical pattern is the first thing to consider, then a tissue biopsy must be performed, leaded with flexible fiberoptic laryngoscopy or direct laryngoscopy. While bronchoscopy is considered the most accurate technique for diagnosis of lesions in the central airways.^13^

Histologically, RRP is composed by papillomatous structures made of abnormal squamous epithelium, where keratinization and basal cell hyperplasia are in excess, with exophytic projections overlying supporting fibrovascular cores. Epithelial atypia is usually absent, although these benign lesions can undergo malignant transformation. Pathological changes include atypia, focal necrosis, foci of keratinization and sheets of polygonal tumoral cells.^5^

Radiological diagnostic strategies include xrays, particularly indicated for RPP with lung involvements; CT scan, that allow to find the presence of focal or diffused airway narrowing on the mucosal surface; MRI, that can detect the presence of lesions in the larynx, tracheobronchial and pulmonary regions. Radiological assessments are necessary to determine the correct staging and, consequently, the correct treatment option.^13,14^

### Treatment

Currently, there is no curative treatment for RRP. However, the primary approach is surgical excision. The aim of the surgical strategy is to debulk the papillomatous lesions at the same time preserving the integrity of the underlying anatomical structures and maintain the airway patency. The excision modalities are multiple and surgeon-dependent; the focus is to prevent damage of surrounding tissue. Recurrences are common, and repeated surgery is often necessary to preserve good respiratory and phonatory quality. For these reasons, double-stage procedures or subtotal resections are preferred, reducing the risks of webbing and scarring.^15^

Different surgical techniques have evolved in the management of RRP, moving from cold instruments and microdebriders to different types of lasers, mainly: ablative/cutting lasers or photoangiolytic lasers.^15^

On the other hand, this strategy can increase the risk of local damage and complications such as laryngeal stenosis, reduction of the respiratory space, formation of tracheoesophageal fistulas and increase expression of HPV dormant in nearby cells.^16^

### Adjuvant treatment

In about 1 out of 5 patients, the disease cannot be controlled by surgery alone and adjuvant treatments are needed. The adjuvant therapy should be considered if palliative surgery is needed more than 4 times a year, in case of rapid recurrence of papillomatous lesions with the risk of airway obstruction and in case of disease spread to the distal respiratory tree. The main purpose of adjuvant treatments is to remodulate the action of the immune system against the effective agent to inhibit the replication of the virus.

Several adjuvant therapies have been administered, with a little consensus on which treatments are most effective and the timing of their administration. Antiviral agents such as interferon-alpha and cidofovir were commonly considered the first line treatment. The mechanism of action is predominantly inhibition of viral nucleic acid synthesis. But the results were heterogeneous and, in some cases, burdened by considerable side effects including neurological disorders, leukopenia and thrombocytopenia.^15,17^

To date, in locally advanced disease or metastatic forms, other therapeutic options taken into account are antiangiogenic monoclonal antibodies, approved as single agent or in combination with chemotherapy, and targeted therapies. Bevacizumab, a recombinant human monoclonal antibody that acts selectively, binding the circulating vascular endothelial growth factor VEGF-A^18^, is one of the agents that have been investigated in these settings.

### Interferon

In the 1980s interferon-alpha was one of the first adjuvant treatments adopted for the treatment of RRP and it was used either intralesional or intramuscular. It is a cytokine that binds to specific cell receptors and modifies the immune response with an anti-proliferative and anti-viral effect.^19^ Its use was progressively abandoned due to the severe side effects, mainly hepatotoxicity.

### Antiviral agents (acyclovir, ribavirin, cidofovir)

The efficacy of Acyclovir and Ribavirin was tested in the 1990s by Bergler *et al*. and in a few case series and seemed to be linked to the presence of viral co-infections (Herpes Syimplex, Cytomegalovirus, or Epstein-Barr virus).^20^ These clinical studies were insufficient to conclude a beneficial effect of these drugs.

Cidofovir on the other hand is a cytosine nucleotide analogue and its introduction resulted in a great improvement in the control of the disease. Once converted in its active form, it is incorporated into DNA and exerts its toxicity in the Papilloma and Herpesviridae families. It can be administered intravenously or intralesional. Unfortunately, systemic treatment has been associated with neutropenia and nephrotoxicity.^21^ The intralesional offlabel use has been adopted for the treatment of genital HPV or RRP, but there are no clear protocols for dose, concentration, and frequency.^22^

Even if many studies have reported significant response rates with almost no side effects in January 2011, a communication provided by the manufacturer of cidofovir addressed very serious side effects concerning its off-label use: reporting nephrotoxicity, neutropenia, oncogenicity and even some fatalities. In 2012 followed a study involving 16 different hospitals in 11 different countries worldwide which submitted 635 RRP patients, of whom 275 were treated with intralesional cidofovir, with no clinical evidence for long-term nephrotoxicity, neutropenia, or malignancies.^19^ Nevertheless, drug import is now allowed only for authorised clinical trials.

**Figure 1.**
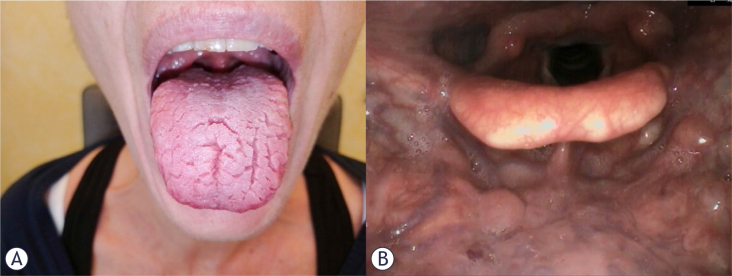
Inspection of the oral cavity and fiberoptic images of patient 1, a 44 yar-old woman, after systemic treatment. **(A)** Picture of tongue fissurization and oral cavity of the patient. **(B)** Oropharyngeal overview of the patient, with no sign of papillomatosis

### Bevacizumab

Bevacizumab is a recombinant monoclonal humanised antibody that blocks angiogenesis by inhibiting human vascular endothelial growth factor A (VEGF-A) and by preventing the activation of its receptor (VEGF-R).^18^ A retrospective study conducted by Rahbar *et al*. demonstrated the role of VEGF-A in the pathogenesis of RRP.^23^ The squamous epithelium of papilloma presented a strong expression of VEGF-A mRNA, and VEGFR-1 and VEGFR-2 were strongly expressed in papilloma’s blood vessels endothelial cells. From these observations Bevacizumab was considered as a treatment. The predominant effect of Bevacizumab in RRP is modulation of vasculature and not the induction of apoptosis, stronger effects are seen coupling the use of this drug with photoangiolytic lasers.^23^

Bevacizumab can be administered both intravenously and intralesionaly, where the intravenous use is indicated for patients with non-accessible lesions, at the dose of 5-15 mg/kg every 2-3 weeks in adults and 5-10 mg/kg every 2-4 weeks in children.^24,25^ Intralesional use instead has an approved dose of 7.5-12.5 mg at 25 mg/ml.^26^

Studies have not yet shown statistically significant differences between the use of intralesional Cidofovir and Bevacizumab, however Bevacizumab shows a higher rate of partial remissions and fewer adverse events.^27^

### Other adjuvant treatments

Other compounds have been proposed, such as celecoxib, indole-3-carbinol, anti-reflux drugs, PD-1 inhibitors, and gefitinib. Unfortunately, no clinical trials are yet available to assess the actual efficacy of these adjuvant treatments.^15^

### Vaccination

Recent research has shown that the HPV vaccine plays a significant role in not only preventing the transmission of RRP but also in aiding the eradication of the disease in an adjuvant context. Currently, two safe and highly immunogenic vaccines are available that effectively stimulate both humoral and cellular immunity: Gardasil, a quadrivalent vaccine containing recombinant HPV proteins targeting genotypes 6, 11, 16, and 18, which aims to prevent cervical and anal cancers^28^, and Gardasil 9, which offers protection against additional HPV genotypes 31, 33, 45, 52, and 58, and is recommended for individuals aged 9 to 45.

Now there are interesting clinical trials ongoing like the INO-3107 (NCT04398433), a DNA immunotherapy designed to elicit targeted T-cell responses against human papillomavirus (HPV) types 6 and 11, in adult patients with recurrent respiratory papillomatosis. Which shows promising results with a tolerable and beneficial effect.^29^

**Figure 2.**
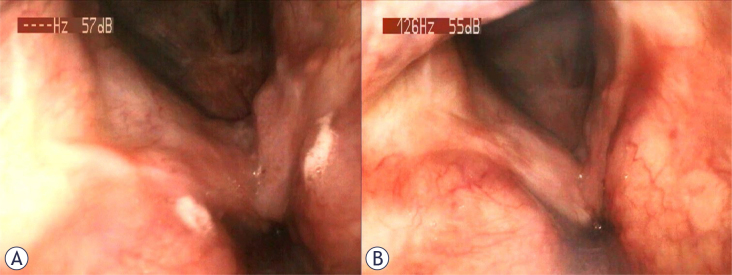
Inspection of the oral cavity and fiberoptic images of patient 2, a 58 years-old male, after systemic treatment. **(A)** Oropharyngeal overview at slightly different angles of the patient **(B)**, with no sign of papillomatosis.

### Presentation of successful treatment cases

We present two cases of RRP successfully treated with surgery, adjuvant systemic Bevacizumab and HPV vaccination.

### Patient 1

A 44-year-old woman, with a previous history of uterus conizations for LSIL (Low grade Squamous Intraepithelial Lesion) / CIN1 (Cervical Intraepithelial Neoplasia) lesions, underwent an ENT evaluation after the onset of widespread papillomatous lesions located on the tip, base and margins of the tongue and on the median raphe of the upper lip.

An excisional biopsy and scraping of the oral cavity were performed. The histological examination of the squamous papillomatous lesions reported the positivity for Human Papillomavirus of genotype 6.

Surgery was evaluated as the best first line treatment option, therefore a bilateral laser vaporization of most of the visible papillomatous lesions on the tip, margins, ventral face, dorsal face and base of the tongue was performed.

Since this surgical treatment resulted in an incomplete removal of the widespread papillomatosis, the case was discussed by our tumor board, and we decided to proceed with systemic adjuvant therapy with Bevacizumab at the dose of 10 mg/kg every three weeks, repeated for six cycles.

At our first oncological evaluation, before the beginning of treatment, the patient was in good clinical conditions, with an ECOG PS equal to 0 and no pathological signs at the physical examination. The inspection of the oral cavity showed papillomatous lesions of the tongue without any symptoms referred.

For each infusion, 470 mg of Bevacizumab were administered. Due to the patient′s previous history of serious allergic reactions both after the administration of the anti-SARS-COV2 vaccine and amoxicillin, an appropriate premedication of hydrocortisone 250 mg and chlorphenamine 10 mg was administered at every cycle. Following the first Bevacizumab infusion the patient reported pain in the right cubital fossa and low-grade edema of the ipsilateral hand with symmetrical isosphygmic peripheral pulses. Deep vein thrombosis was suspected; but the venous doppler ultrasound scan of the arms resulted negative. The edema then resolved spontaneously. Furthermore, after the third infusion, an isolated case of proteinuria G1 was detected at urine analysis. The treatment was overall well tolerated with no clinically significant side effects reported.

After the treatment completion, at the first oncological re-evaluation the patient complained of sore throat and foreign body sensation. At the oral cavity examination, diffuse papillomatous lesions in the left edge, lingual base and right labial fornix were observed, although decreased in number and locations; hypertrophy of the right tonsil was also noticed. New biopsies and scraping of the oral cavity were performed. On the right anterior lingual margin and on the left posterior lingual margin, squamous papillomas with diffuse superficial erosion were diagnosed. The Innogenetics tests performed on the scrapings of the oropharynx resulted as negative, both for the identification of HPV DNA and HPV genotypes.

The patient performed a facial MRI, which reported the absence of signal alterations affecting the lingual body or suspicious DWI signal abnormalities; only some immunoreactive lymph nodes at the IIa level of the right neck were highlighted (the largest one of 11 × 7 mm of diameter).

Three months after the completion of treatment with Bevacizumab, the patient underwent the anti-HPV vaccination for therapeutic purposes and two doses of Gardasil 9 were administered without remarkable side effects.

The patient will undergo an annual ENT follow-up.

### Patient 2

A 58-year-old male, with no significant pathological anamnesis (GERD, disc herniation), underwent ENT evaluation due to breathing difficulty and hoarseness; turbinates′ hypertrophy and vegetative exophytic neoformation on the left epiglottis were diagnosed, so the specialist recommended the turbinates′ reduction with radiofrequency and the neoformation′s removal. The patient underwent both procedures, and the histological examination came back positive for HPV-related squamous cells laryngeal papillomatosis, p16-, low risk strains (genotyping not executable). Three months later, a new episode of dysphonia occurred; again, ENT evaluation was requested, and the reappearance of papillomatous lesions was diagnosed. The patient then underwent a second exeresis (CO2 laser mediated) of a right arrhythmenoideal neoformation and a left anterior commissural lesion. To histological examination: HPV-related squamous cells papillomatosis; HPV-DNA positive for lowrisk strains. Two months later, at ENT follow-up re-evaluation, the patient was diagnosed with a recurrence of the disease (appearance of: four laryngeal papillomatosis lesions, one infrahyoid lesion, one right false vocal cord lesion, one left true vocal cord lesion conditioning phonatory difficulty). He then underwent HPV-vaccination (3 inoculations) and surgical exeresis of the above-mentioned lesions, again with histological positivity for HPV.

Because of the evidence of a new recurrence of disease on the left vocal cord one month later, the patient was candidate to systemic treatment with intravenous Bevacizumab, at the dose of 10 mg/kg q21 for 6 cycles, after which an instrumental restaging with CT scan will be carried out. The treatment was overall well tolerated; drug-induced hypertension (maximum values of 140/90 mmHg) responsive to low dose antihypertensive, and occasional itching well responsive to low doses of antihistamines were observed.

Due to the appearance of leukoplakia e aphthous stomatitis of the oral cavity, the patient underwent local ozone therapy treatment sessions with clinical benefit.

The CT scan performed at the end of the treatment administration reported the complete resolution of the two lesions on the left vocal cord previously described and the total absence of new ones. The patient will undergo an annual ENT follow-up.

## Discussion

In 2009 Nagel *et al*. described a case of pulmonary and tracheal RRP requiring laser-debridement 4 times a year over a 10-year period.^30^ The patient had a significant regression of the disease following the first systemic administration of Bevacizumab. In 2017, after many years of clinical experimentation with a nationwide survey, Best *et al*. concluded that systemic Bevacizumab at a dose of 5–10 mg/kg every 2–4 weeks showed significant positive outcome in patients with advanced, treatment-resistant papillomatosis.^31^ The reason behind these results is that RRP lesions possess a vascular nature, therefore drugs designed to disrupt blood vessels′ density or formation are effective in hindering papillomatous lesions′ progression. Bevacizumab, being an anti-VEGF monoclonal antibody, administered both locally (intralesional injection) or systemically (intravenous injection) has shown excellent results.^32^

The major concern about the use of an antiangiogenic therapy is the rebound papilloma′s growth within the cessation of the treatment, since VEGF-blockade alone does not intrinsically activate immunity against HPV. Moreover, Bevacizumab has a known side effect profile (renal insufficiency), and prospective studies are needed to determine optimal long-term dosing schedules aimed at keeping enough drug in the system to suppress papilloma′s growth while reducing the risk of adverse events. An interesting solution could be the concomitant use of immunotherapy, to both reduce the burden of the residual disease and activate the immune system against the HPV infected cells. These aspects certainly deserve further studies. Promising data concern the possible use of HPV vaccine in the adjuvant treatment of RRP, despite the primary purpose of vaccination is to prevent future HPV infection. In their study, Young *et al*. showed a clinical benefit of HPV vaccination in RRP patients: eight patients experienced complete remission, and five patients experienced partial remission.^28^ Also, Yiu *et al*. demonstrated an increase in the time interval between repeated surgeries in vaccinated patients.^33^ The mechanism that underlies the vaccine′s potential therapeutic efficacy against HPV is still unknown.

Therefore, two strategies against RRP should be considered: firstly, the vaccine reduces the risk of infection and consequently the incidence of HPV-related pathologies; secondly, patients who tested anti-HPV positive at baseline could develop a booster response to vaccination that prevents the infection from recurring.^34^ For these reasons HPV vaccination is the most promising development in the treatment of RRP.^15^

Despite the encouraging literature on adjuvant HPV vaccination for secondary prevention in RRP, this strategy has not yet been accepted widely in treating the RRP population: the inconsistent findings data from published reports underline the need for evaluation of therapeutic efficacy of currently available HPV vaccines for the management of RRP.

## Conclusions

RRP is a chronic disease currently difficult to treat due to the unpredictability of its recurrences and aggressive nature. The cases we reported are an example of the efficacy of systemic Bevacizumab at the dose of 10 mg/kg as a first line adjuvant therapy. As previously published clinical cases and series suggest, this treatment has shown a safe profile with overall good results, with the shortterm local eradication of HPV. Further randomized prospective research with long-term follow-up is needed to better define the safety of this agent and the results against the disease′s recurrence.

We also discussed the effectiveness of anti-HPV vaccination, not only as a prophylaxis of the infection but also as adjuvant treatment in preventing or delaying the recurrence of the disease, a role that needs to be demonstrated with further studies.
